# M13 phages engineered with chlamydia phage φCPG1 protein IN5 and arginine-glycine-aspartic acid inhibits *Chlamydia trachomatis* intracellular growth

**DOI:** 10.1016/j.virusres.2025.199645

**Published:** 2025-10-18

**Authors:** Cong You, Mei Wang, Jiangyi Wang, Tingting Lian, Quanzhong Liu

**Affiliations:** a Department of Dermatovenereology, Tianjin Medical University General Hospital, No. 154 Anshan Road, Heping District, Tianjin 300052, China; b Department of Dermatology and Venereology, The First Affiliated Hospital of Gannan Medical University, No. 23 Qingnian Road, Zhanggong District, Ganzhou 341000, China

**Keywords:** *Chlamydia trachomatis*, Inhibition, M13 phage, Arginine-glycine-aspartic acid, IN5, Recombinant phage

## Abstract

*Chlamydia trachomatis* (*C. t*) is the most common causative agent of sexually transmitted bacterial urogenital infections worldwide. *C. t* treatment failure is increasing because antibiotic resistance has developed in recent years. Therefore, the development of novel therapeutic strategies is necessary. Here, we constructed an M13 phage carrying two functional peptides, including the integrin binding peptide arginine-glycine-aspartic acid (RGD) on pⅧ and the IN5 protein from *Chlamydia caviae* phage φCPG1 on pIII to reduce *C. t* infection. We called these phages M13-RGD_8_-IN5_3_. The recombinant phages successfully expressed IN5 proteins. Confocal laser scanning microscopy confirmed that the recombinant phages were able to enter HeLa cells and *C. t* inclusion bodies. IN5 protein was responsible for the observed decrease in *C. t* infection, while RGD enhanced the permeability of phages into the cells. The M13-RGD_8_-IN5_3_ phage was better than the M13-IN5_3_ phage in ameliorating *C. t* infection. qPCR revealed that treatment with the recombinant phages downregulated several *C. t* genes related to virulence, such as *CT_046* (*Hc2*), *CT_443* (*OmcB*), *CT_444* (*OmcA*), *CT_456* (*Tarp*), *CT_666* (*Cdsf*), *CT_694, CT_743* (*Hc1*)*, and CT_875* (*TepP*). The only upregulated gene was *CT_119* (*IncA*)*.* The recombinant phages impacted the *C. t* mainly in the middle and late stages of the development cycle. Our results suggest that novel recombinant phages are promising as candidates to treat *C. t* infection.

## Highlights

• The recombinant M13-RGD8-IN53 could significantly reduce C. t infection.
• The M13-RGD8-IN53 phage was better than the M13-IN53 phage in ameliorating C.t infection.
• Treatment with the recombinant M13-RGD8-IN53 and M13-IN53 phages downregulated C. t genes related to virulence.

## 1 Introduction

*Chlamydia trachomatis* (*C. t*) is the most common causative agent of bacterial sexually transmitted infections worldwide. According to a news released on November 21st, 2024, World Health Organization estimates that in 2020, an estimated 128.5 million new infections with *C. t* occurred worldwide among adults aged 15 to 49 years. The global prevalence among people aged 15–49 years was estimated to be 4.0 % for women and 2.5 % for men in 2020 (World Health Organization, 2025). According to the European Centre for Disease Prevention and Control, in 2022, the number of reported cases saw a significant increase compared to the previous year, with *C. t* cases by 16 % in Europe (European Centre for Disease Prevention and Control, 2025). In China, the reported incidence rates of genital *C. t* infection was 12.45 per 100 000 in 2023 (Jian et al., 2014). The *C. t* D to K serotypes may lead to cervicitis in females and urethritis in males. Persistent infection of *C. t* without symptoms is associated with some sequelae and complications, including pelvic inflammatory diseases, infertility, and ectopic pregnancy in female patients (Tsevat et al., 2017).

*C. t* is an obligate intracellular bacterium that has a special biphasic life cycle comprised of a replicative non-infectious reticulate body (RB) and an infectious elementary body (EB) (Stephens et al., 2011). Both forms develop in a membrane-bound compartment, which is called an inclusion, within host cells. The developmental cycle of *C. t* requires approximately 48 h and is divided into three different stages: early (0–12 h), mid (12–24 h), and late (24–48 hours) stages (Brockett and Liechti, 2021). There are various reports describing the emergence of antibiotic resistance, particularly to erythromycin and doxycycline. Treatment failure in clinical practice has been documented in human urogenital tract infections (Manhart et al., 2013). In addition, treatment failure still exists despite changing treatment regimens or combined treatments, indicating that *C. t* may have developed antibiotic resistance via drug-resistant gene mutations (Shao et al., 2020). Therefore, it is necessary to develop new treatment methods with novel mechanisms to overcome the current treatment challenges. Until now, little progress has been made in developing a vaccine for *C. t.* Thus, the use of specific Chlamydia phages may be an alternative solution. Six bacteriophages have been isolated from Chlamydiae: Chp1, Chp2, Chp3, Chp4, φCPG1, and φCPAR39. All Chlamydia phages share similar molecular characteristics. However, no phages have yet been isolated from *C. t.* φCPG1 is a lytic phage specific to *Chlamydia caviae*, which is a natural parasite of the guinea pig. The φCPG1 genome encodes Vp1, Vp2, and Vp3 capsid proteins. Among them, Vp1 plays essential roles in host cell adhesion and invasion. We previously showed that Vp1 inhibits *C. t* growth via the mitogen-activated protein kinase (MAPK) pathway (Guo et al., 2016). Insertion loop 5 (IN5), which forms a mushroom-like protrusion in Vp1, is a special region on the surface of Chlamydia phage that plays a key role in host recognition. We successfully expressed and purified IN5 protein and confirmed its inhibitory effect on *C. t* growth, with an inhibition rate of 54.50 % (Zheng et al., 2017). In addition, we found that polymorphic membrane protein I (PmpI) of *C. t* interacts with Vp1 and might be a potential Vp1 receptor that inhibits *C. t* (Ren et al., 2018). However, because *C. t* is an obligate intracellular bacterium, interactions between Vp1 or IN5 and PmpI are reduced after it enters the host cells. Further, the mechanism of IN5 inhibition related to *C. t* invasion has not been explored.

Several lytic or virulent phages such as T1 to T7 phages have been developed for “phage therapy” to treat human drug-resistant bacterial infectious diseases such as salmonellosis, acute and chronic urogenital inflammation, skin ulcers, dysentery, and post-surgical wounds (Kutter et al., 2010; Abedon et al., 2011). Temperate phages cannot lyse host cells and are unsuitable candidates for natural phage therapy. These phages may display active ingredients for targeted therapeutic delivery. The M13 phage is a temperate phage that is often used in phage engineering and has been successfully applied *in vivo*. Importantly, its application has been approved by the U.S. Food and Drug Administration. There are no detrimental effects of M13 phages on mammalian cells (Lang, 2006; Atterbury, 2009; Donnelly et al., 2015). M13 phage vectors can contain more than 2700 peptides and are efficient for gene delivery (Pranjol and Hajitou, 2015). The capsid proteins, such as pⅧ, covering M13 phage surface enable delivery of multiple therapeutic peptides or proteins such as the integrin binding peptide arginine-glycine-aspartic acid (RGD). RGD is effective in inducing integrin-mediated endocytosis, which leads to high cellular attachment of RGD-coated M13 phages (Safari et al., 2019). M13-RGD bacteriophages have been exploited alone or in combination with peptides or proteins (Shin et al., 2014; Yoo et al., 2016; Jin and Lee, 2018; Chae et al., 2019). Bhattarai et al. developed an engineered M13 phage that expresses two functional peptides–RGD and *C. t-*associated polymorphic membrane protein D (pmpD)–to ameliorate *C. t* infection (Bhattarai et al., 2012). The RGD peptide and *C. t pmpD* protein were expressed on the pⅧ major and pIII minor coat proteins, respectively, of the M13 phage. In our previous studies, we found that IN5 protein effectively inhibits *C. t* propagation. In addition, we successfully constructed a M13-IN5_3_ (φCPG1 protein IN5 on pIII) recombinant phage that expresses IN5 protein and enters the inclusion body of *C. t* and inhibits its growth (Lian et al., 2018). However, the specific mechanism underlying this inhibitory effect has not yet been explored. In our study, we hypothesized that a modified M13-RGD_8_-IN5_3_ (RGD peptide on pⅧ and φCPG1 protein IN5 on pIII) phage was better than the M13-IN5_3_ phage in reducing *C. t* infection.

In the present study, we engineered an M13-RGD_8_-IN5_3_ phage to improve *C. t* inhibition compared with M13-IN5_3_ and IN5. Then, we investigated the effects of the recombinant phage on *C. t* in the early, middle, and late stages. We also measured the transcription of several chlamydial genes related to its virulence to investigate the underlying mechanism of the inhibitory effect on *C. t.* This new phage could constitute a new strategy to reduce *C. t* infection.

## 2 Materials and methods

### 2.1 Construction and identification of recombinant M13-RGD_8_ and M13-RGD_8_-IN5_3_ phages

Primers (Table 1) were designed according to the M13 (**NCBI GenBank: L08821.1**), RGD (**NCBI Gene ID: 24,511**), and IN5 (**NCBI Gene ID: 1261,929**) sequences, and were synthesized by GENEWIZ Biotech company, China (Table 1). We used the same primers to construct the recombinant M13-RGD_8_ and M13-RGD_8_-IN5_3_ phages. M13 (New England Biolabs, Ipswich, MA, USA) and M13-IN5_3_ phages (stored at the Tianjin Institute of Sexually Transmitted Diseases, China) were used as templates (Lian et al., 2018). The linear sequences were amplified by PCR, purified on agarose gels, and eluted with spin column purification (E.Z.N.A.® Gel Extraction Kit, Omega Bio-tek, Norcross, GA, USA). The PCR procedure was as follows: one cycle of 3 min at 94 °C followed by 30 cycles of 30 s at 94 °C, 30 s at 55 °C, and 1 min at 72 °C, with a final extension step at 72 °C for 5 min. The PCR products were assembled with an In-Fusion cloning kit (Takara, Dalian, China). The fusion product was transformed into competent *Escherichia coli* K12 ER2738 (New England Biolabs, Ipswich, MA, USA) during the exponential growth stage. Three milliliters of 0.8 % (w/v) agar (45 °C) were poured into pre-warmed LB/isopropyl-beta-d-thiogalactopyranoside (IPTG)/tetracycline plates. The transformed *E. coli* were then added onto the plates, which were incubated overnight at 37 °C for phage proliferation. The plates were observed the next day and several plaques were sent for gene sequencing by GENWIZ Biotech (Suzhou, China) to verify that the bacterial colonies contained the correct M13-RGD_8_ and M13-RGD_8_-IN5_3_ sequences. The resulting M13-RGD_8_ and M13-RGD_8_-IN5_3_ phages were transformed into competent *E. coli* for subsequent proliferation.

**Table 1:** Primers used for pVIII engineering.

Product name	Forward primer (5′→3′)	Reverse primer (5′→3′)
M13-RGD-IN5 P1	GGTATTTCCATGAGCGTTTTTCC	GGGATCTTCGGTGTCACCACGTCCCAAGTCTGCAGCGAAAGACAGCATCGGAAC
M13-RGD-IN5 P2	GGAAAAACGCTCATGGAAATACC	GCAGACTTGGGACGTGGTGACACCGAAGATCCCGCAAAAGCGGCCTTTAAC

### 2.2 Proliferation and concentration of recombinant M13-RGD_8_ and M13-RGD_8_-IN5_3_ phages

The *E. coli* which was successfully transformed with recombinant phages were inoculated in the Luria-Bertani culture medium, which were incubated overnight at 37 °C for proliferation. The suspension was centrifuged at 14,000 g for 15 min at 4 °C, the precipitation was discarded and the supernatant was centrifuged at the above parameters again, then 2 mL polyethylene glycol (PEG)/sodium chloride (NaCl) (Solarbio, Beijing, China) solution was added to the supernatant and incubated overnight at 4 °C. The next day the suspension was centrifuged at 25,000 g for 10 min at 4 °C. The precipitation was resuspended with 1 mL normal saline and 200μ L PEG/NaCl solution, then the suspension was centrifuged at 25,000 g for 10 min at 4 °C. The precipitation containing phages was left and resuspended with normal saline for further proliferation and concentration.

### 2.3 IN5 protein analysis by western blotting

Recombinant phage proteins were extracted using a radioimmunoprecipitation assay with protease inhibitor (Solarbio, Beijing, China). Total proteins were separated by 10 % sodium dodecyl sulfate polyacrylamide gel electrophoresis (SDS-PAGE) and transferred onto polyvinylidene fluoride (PVDF) membranes (Millipore Immobilon-P, Darmstadt, Germany). The membranes were incubated overnight with rabbit anti-IN5 polyclonal antibodies (1:1000; stored at Tianjin Institute of Sexually Transmitted Diseases, China). Purified IN5 protein (stored at Tianjin Institute of Sexually Transmitted Diseases) was used as a positive control. Then, goat anti-rabbit IgG (*H* + *L*)–HRP (1:5000; ZSGB Biotech Cor. Beijing, China) was added to the membranes. The PVDF membranes were imaged using a Tanon 5200 Gel Imaging System (Tanon Cor. Shanghai, China) after development using an electrochemiluminescence solution (Absin, Shanghai, China).

### 2.4 Quantification of viable recombinant M13 phages

First, 200 μL of stored phages was diluted in sterile LB culture medium in a 10-fold serial dilution ranging from 10^9^ pfu/mL to 10^12^ pfu/mL. Then, 10 μL of phages in each dilution was added to 200 μL LB medium containing *E. coli* ER2738 at the exponential phase. The mixture was incubated for 10 min at 25 °C. *E. coli* with phages were added onto agar plates and cultured as previously described. The plates were inspected the next day and plaques were counted. The phage titer was determined by plaque assay using plaque-forming units per milliliter (pfu/mL) = plaque counts × dilution factor × 100. The phages were temporarily stored in normal saline solution at a titer of 10^11^ pfu/mL at 4 °C.

### 2.5 C. t D UW-3/CX culture and immunofluorescence microscopy

HeLa-229 cells were obtained from the Type Culture Collection of the Chinese Academy of Sciences (Shanghai, China). Cells were cultured in Dulbecco’s modified Eagle’s medium (DMEM; HyClone; GE Healthcare Life Sciences, Logan, UT, USA) supplemented with 10 % fetal bovine serum (FBS, Haoyang, Tianjin, China) at 37 °C in a humidified 5 % CO_2_ incubator using 24-well plates with coverslips. The reference *C.* t D UW-3/CX strain was purchased from the American Type Culture Collection and stored in sucrose-phosphate-glutamate buffer at −80 °C. HeLa cells (4 × 10^5^) grown in a monolayer in 24-well plates were treated for 30 min with 30 μg/mL diethylaminoethyl dextran to increase infectivity. *C. t* cells at an MOI of 1 were added to the HeLa cells, and the plates were centrifuged at 1200 *g* for 1 h at 32 °C. Then, the cells were incubated for 2 h at 37 °C in a 5 % CO_2_ incubator. After incubation, the supernatant was aspirated, and the infected monolayers were overlaid with culture medium supplemented with 10 % FBS and 1 μg/mL cycloheximide (Bailingwei Technological Co., Ltd., Beijing, China). The plates were incubated at 37 °C in 5 % CO_2_ for 12–48 h. HeLa cells infected by *C. t* were mounted on glass coverslips, washed with PBS, and fixed with methanol for 15 min. The fixed cells were treated with 0.1 % (w/v) Triton-X-100 (Solarbio, Beijing, China) for 8 min at 25 °C and blocked in 10 % (w/v) FBS for 1 h at 37 °C. The cells were then incubated with polyclonal rabbit antibody against the *C. t D* serotype lipopolysaccharide (obtained from Professor Guangming Zhong, University of Texas, San Antonio, TX, USA) in 10 % FBS at 4 °C overnight. Following three washes in PBS, the monolayers were incubated with FITC-conjugated goat anti-rabbit antibodies (1:200; Abcam, Cambridge, UK) in 10 % FBS and 10 μg/mL DAPI (blue) for 1 h at 37 °C. Images were acquired using a Zeiss Axio Imager A2 fluorescence microscope. All experiments were performed in triplicate.

### 2.6 Species- and dose-dependent inhibitory effects of recombinant phages on C. t proliferation

Before inoculation into HeLa cells, 10^6^–10^9^ pfu/mL of M13, M13-RGD_8_, M13-IN5_3_, and M13-RGD_8_-IN5_3_ phages were co-incubated with *C. t* for 3 h. *C. t* infection alone was used as a control. The design of the treatment groups is presented in Table 2. The inoculation procedure for *C. t* was performed as described previously. At 48 h post-infection (p.i.), *C. t* was re-cultivated in HeLa cell monolayers in a progeny infectivity assay. Briefly, the infected HeLa cells were disrupted by ultrasonication and centrifuged at 1200 *g* for 5 min. Then, the supernatants were used to infect fresh monolayers of HeLa cells to determine the infectivity of EB. To determine the optimal titer of each recombinant phage for inhibition of *C. t* growth, quantitative analysis of inclusion numbers per visual field at 100 × magnification was performed 48 h p.i.

**Table 2:** Experimental groups for species-dependent effects Using different phages.

Groups	Control	M13	M13-RGD_8_	M13-IN5_3_	M13-RGD_8_-IN5_3_
HeLa cell	√	√	√	√	√
*C. t*	√	√	√	√	√
M13		√			
M13-RGD_8_			√		
M13-IN5_3_				√	
M13-RGD_8_-IN5_3_					√
Normal saline	√				

### 2.7 CCK-8 assays

Cell Counting Kit-8 (Dojindo Molecular Technologies, Inc., Shanghai, China) was used to assess cell viability. HeLa cells (5000) were seeded and incubated in 96-well plates with or without 10 μL phages at different titers (0, 5 × 10^9^, 10 × 10^9^, 15 × 10^9^, 20 × 10^9^, or 25 × 10^9^ pfu/mL). Three replicates were used in each group. The culture plates were incubated at 37 °C in a humidified 5 % CO_2_ atmosphere for 48 h. Then, CCK-8 reagent (10 µL) was added to each well. After incubation for 4 h at 37 °C, the absorbance of the medium was read at 450 nm using a microtiter plate reader (Bio-Rad Laboratories, Inc., USA). HeLa cells without phages were used as a control.

### 2.8 Distribution of phages in C. t-infected HeLa cells observed by confocal laser scanning microscope

To test phage uptake, different phages at a titer of 10^9^ pfu/mL were co-incubated with *C. t* (MOI = 1) and inoculated into HeLa cells. *C. t* without phages was used as a control. After 48 h, inclusions in HeLa cells were immunostained as previously described. The primary antibodies were polyclonal mouse antibody against the *C. t* D serotype lipopolysaccharide (obtained from Professor Guangming Zhong, University of Texas, San Antonio, TX) and rabbit M13 phage-specific monoclonal antibody (1:2000; Anti-fd bacteriophage; Sigma). The secondary antibodies were FITC-conjugated goat anti-mouse antibodies (1:200; Abcam) and goat anti-rabbit IgG–AlexaFluor 594 (1:100; ZSGB Biotech Cor., Beijing, China) in 10 % FBS. Nuclei were stained with 10 μg/mL DAPI. Cells were incubated with secondary antibodies for 1 h at 37 °C. Images were acquired using a LSM880 confocal laser-scanning microscope (Zeiss).

### 2.9 Immunofluorescence microscopy and quantitative polymerase chain reaction to analyze the effect of engineered phages on C. t infection

Before inoculation into HeLa cells, 10^9^ pfu/mL M13-IN5_3_ and M13-RGD_8_-IN5_3_ phages were co-incubated with *C. t* for 3 h. *C. t* infection alone was used as a control. The inoculation procedure for *C. t* was performed as described in 2.4. Semi-quantitative analysis of mean fluorescence intensities of inclusion per field was performed using NIH Image J software (version 1.52; National Institutes of Health*,* Bethesda, MA, USA) at 12, 24, 36, and 48 h p.i. Inclusion numbers per visual field at 100 × magnification were calculated at 24, 36, and 48 h p.i. To determine the presence and titers of phages in the HeLa cells, the cells were collected at 12, 24, 36, and 48 h p.i., resuspended in normal saline, disrupted, and passed through 0.22-μm filters (Millipore, Merck Millipore Ltd, MA, USA). The filtrate was used for phage quantification as 2.3 described.

Total RNA was extracted from HeLa cells with *C. t* and phages at 12, 24, 36, and 48 h p.i. using a FastPure® Cell/Tissue Total RNA Isolation Kit V2 (Vazyme Biotech Co., Nanjing, China). Then, 1 µg total RNA was used to synthesize first-strand cDNA in a 20-µL reaction using HiScript III RT SuperMix for qPCR (+ gDNA wiper) (Vazyme Biotech Co., Nanjing, China). The cDNA product (1 µL) was used for quantitative PCR with ChamQ Universal SYBR qPCR Master Mix (Vazyme Biotech Co., Nanjing, China) and a Cobas z 480 system (Roche, Basel, Switzerland). The primers were synthesized by Sangon Biotech Co., Ltd. (Shanghai, China; Supplementary Table S1). The PCR procedure was as follows: one cycle of 30 s at 95 °C followed by 40 cycles of 10 s at 95 °C and 30 s at 60 °C, followed by melting curve analysis at 95 °C for 15 s, 60 °C for 60 s, and 95 °C for 15 s. The results were analyzed using the 2^−ΔΔct^ method, and *CT_r01*(16SrRNA) was used as the internal reference. All experiments were performed in triplicate. *C. t* without phages was included as a control.

### 2.10 Data analysis

The data were analyzed using GraphPad Prism 9.0 (Insightful Science Company, San Diego, CA, USA). Two-tailed Student’s *t*-tests were used to analyze differences between two groups. One-way ANOVA followed by Bonferroni’s multiple comparison test was used to determine differences between multiple groups. Data are presented as the mean ± standard deviation; *p* < 0.05 was considered statistically significant.

## 3 Results

### 3.1 Genetic engineering the M13 phage

**Fig. 1:**
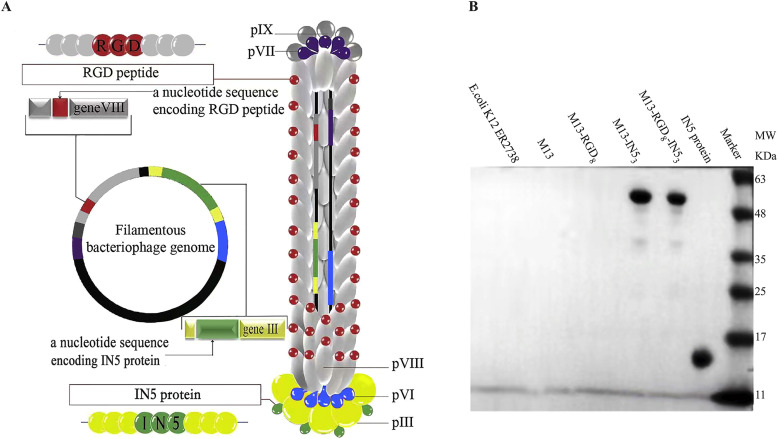
(A) Schematic of the M13 phage peptide library and gene construction. The phage expresses high density signaling RGD motifs on pⅧ and IN5 protein on pⅢ. (B) Western blotting of IN5/pⅢ fusion proteins. The molecular weights of IN5 and IN5/pIII fusion protein were 14 and 58 kDa, respectively.

To generate a recombinant phage, we amplified and fused DNA segments encoding M13, RGD, and IN5 to generate the appropriate plasmids. The engineered M13 phage displayed the desired fusion proteins or peptides on the phage surface. RGD peptides on pⅧ (termed M13-RGD_8_ and M13-RGD_8_-IN5_3_) were designed to enhance phage endocytosis by HeLa cells, while *C. caviae* phage protein IN5 on pⅢ (termed M13-IN5_3_ and M15-RGD_8_-IN5_3_) were designed to inhibit *C. t* growth (Fig. 1A).

### 3.2 Confirmation of IN5 protein expression and recombinant phage quantification

**Fig. 2:**
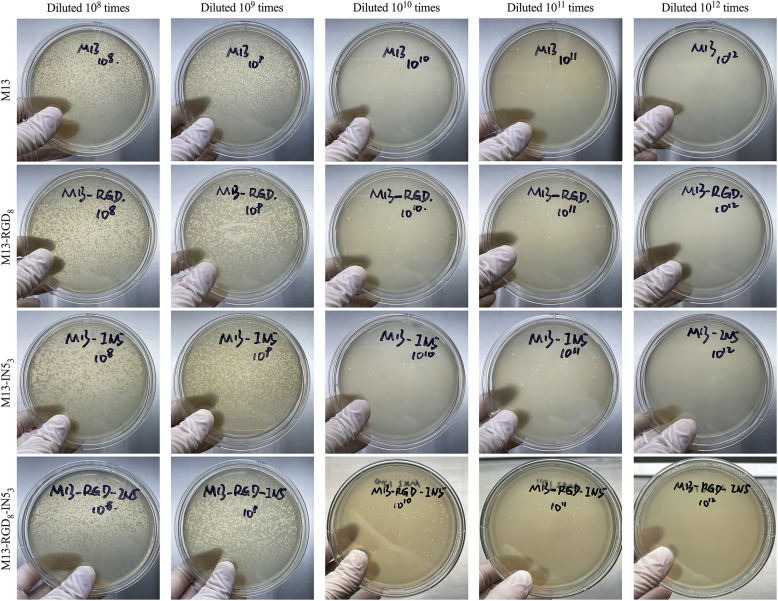
Viral plaques formed by engineered phages. The recombinant phages were cultured and quantified by plaque-forming units per milliliter (pfu/mL) to confirm their viability and titers.

Supplementary Fig. S1 shows how the plasmids were developed for M13-RGD_8_-IN5_3_ and M13-RGD_8_ phage construction. Supplementary Fig. S2 shows the DNA sequences and expression locations in the M13-RGD_8_ and M13-RGD_8_-IN5_3_ phages. IN5 expression was confirmed by western blotting (Fig. 1B). According to the plaque assays, the titers of M13, M13-RGD_8_, M13-IN5_3_, M13-RGD_8_-IN5_3_ phages were 1.6 × 10^14^ pfu/mL, 6.1 × 10^14^ pfu/mL, 3.3 × 10^14^ pfu/mL, and 1.4 × 10^14^ pfu/mL, respectively (Fig. 2).

### 3.3 Species and dose-dependent effects of M13-IN53 and M13-RGD8-IN53 on C. t proliferation and cytotoxicity in HeLa cells

**Fig. 3:**
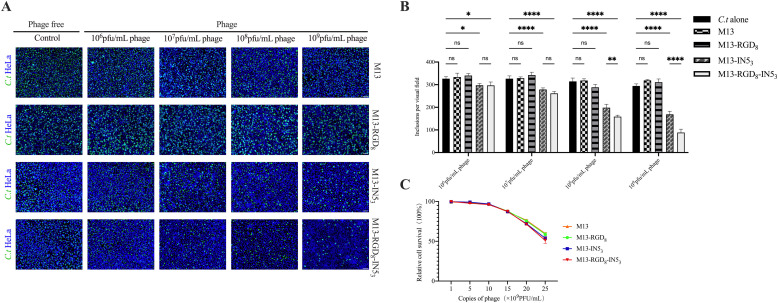
Species and dose-dependent effects of engineered phages on *C. t* proliferation and HeLa cells. (A) *C. t* at an MOI of 1 were co-incubated with different titers of each phage. *C.t* infection alone was used as a control. The first generation of *C. t* cultures was collected and re-inoculated into HeLa cells. The number of inclusions in the HeLa cells was visualized and counted by fluorescence microscopy after 48 h. Scale bar = 50 μm; Blue: DAPI; Green: chlamydia inclusions. (B) Progeny infectivity assays showed that compared with the control group, there was no significant difference of the inclusion numbers in the M13 group and M13-RGD_8_ groups at 48 h p.i. (ns, *p* > 0.05). The number of inclusion bodies decreased significantly in the M13-IN5_3_ and M13-RGD_8_-IN5_3_ groups at titers ranging from 10^6^ pfu/mL to 10^9^ pfu/mL (*p < 0.05; ****p < 0.0001). Increased phage titer significantly decreased inclusion bodies, especially in the M13-RGD_8_-IN5_3_ group (**p < 0.01; ****p < 0.0001). (C) Cytotoxicity of M13, M13-RGD_8_, M13-IN5_3_ and M13-RGD_8_-IN5_3_ at different titers including 0, 5 × 10^9^ pfu/mL, 10 × 10^9^ pfu/mL, 15 × 10^9^ pfu/mL, 20 × 10^9^ pfu/mL, and 25 × 10^9^ pfu/mL in HeLa cells. [Cell survival rate = (experimental well OD-blank OD) / (control well OD- blank OD) × 100 %]. Data represent the mean ± standard deviation of three independent experiments.

HeLa cells with *C. t* were treated with phages that were 10-fold serially diluted from 10^6^ pfu/mL to 10^9^ pfu/mL. Untreated *C. t*, the M13 phage, and M13-RGD_8_ (with only RGD on pⅧ) were used as controls. We determined that infection with M13-IN5_3_ and M13-RGD_8_-IN5_3_ phages were able to inhibit *C. t* proliferation (*p* < 0.05, *p* < 0.0001, Fig. 3A, 3B), while the M13 and M13-RGD_8_ phages did not have any inhibitory effects (*p* > 0.05, Fig. 3A, 3B). According to the number of inclusions per visual field in the progeny infectivity assay, the inhibition was dose-dependent, with greater inhibitory effects when the phage titers increased. The 10^9^ pfu/mL titer of M13-IN5_3_ and M13-RGD_8_-IN5_3_ phages exhibited the most significant inhibitory effects (*p* < 0.0001, Fig. 3A, 3B, Supplementary Table S2). Compared with the M13-IN5_3_ phage group, the M13-RGD_8_-IN5_3_ phage group inhibited *C. t* proliferation more significantly at titers of 10^8^ and 10^9^ pfu/mL (*p* < 0.01, *p* < 0.0001, Fig. 3B). The inhibitory rates were 36.44 % and 52.00 % at 10^8^ pfu/mL for M13-IN5_3_ and M13-RGD_8_-IN5_3_, respectively, and the inhibitory rates were 48.09 % and 69.79 % at 10^9^ pfu/mL for M13-IN5_3_ and the M13-RGD_8_-IN5_3_, respectively (Supplementary Table S2). CCK-8 assays were used to evaluate the cytotoxic effects on HeLa cells. Fig. 3C shows that above 10^9^ pfu/mL, the relative cell survival rate decreased compared with untreated HeLa cells, which confirmed that phage titers less than or equal to 10^9^ pfu/mL did not induce cytotoxic effects. This finding suggests that the recombinant phages at appropriate titers are not cytotoxic. Therefore, we used recombinant phages at a titer of 10^9^ pfu/mL in subsequent experiments.

### 3.4 Validation of phage localization

**Fig. 4:**
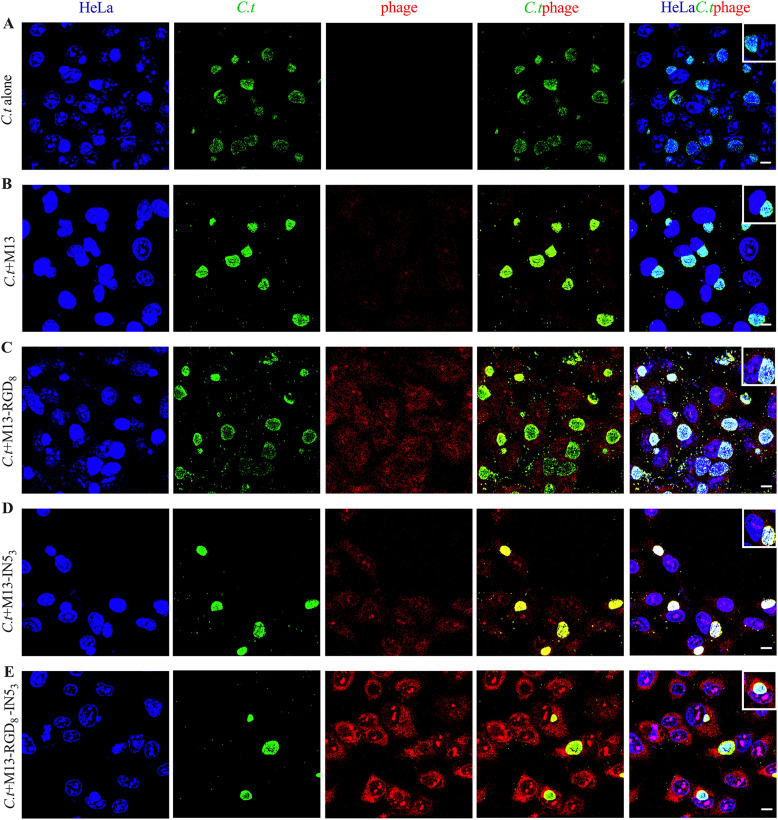
Representative images of phage distribution in *C.t-*infected HeLa cells treated with M13, M13-RGD_8_, M13-IN5_3_, and M13-RGD_8_-IN5_3_ was observed by confocal laser scanning microscopy. (A) HeLa cells were infected with *C. t* serovar D (MOI=1) and fixed at 48 h post-infection (p.i.). *C. t* infection alone was used as a control. (B) Pre-treating with 10^9^ pfu/mL M13 phage for 48 h indicated few phages in HeLa cells. (C) Pre-treatment with 10^9^ pfu/mL M13-RGD_8_ phage for 48 h showed more phages in HeLa cells, but not in the inclusion lumens, compared with the M13 phage group. (D) Pre-treatment with 10^9^ pfu/mL M13-IN5_3_ phages for 48 h showed some phages in HeLa cells and a few of them moved into the inclusion lumen. (E) Pre-treatment with 10^9^ pfu/mL M13-RGD_8_-IN5_3_ phage for 48 h showed many phages in HeLa cells, and a large number of phages entered the inclusion lumen. Blue: DAPI; Green: *C. t* inclusions; Red: phages. Scale bar = 10 μm; Images are representative of three independent experiments.

As shown in Fig. 4, after 48 h p.i., HeLa cells with *C. t* and phages were analyzed to determine phage localization. M13, M13-RGD_8_, M13-RGD_8_-IN5_3_, and M13-IN5_3_ could enter and accumulate around HeLa cell nuclei. Of these, only M13-RGD_8_-IN5_3_ and M13-IN5_3_ could translocate into the inclusion lumen. There were more M13-RGD_8_ and M13-RGD_8_-IN5_3_ phages in HeLa cells than M13 and M13-IN5_3_ phages. Additionally, there were more M13-RGD_8_-IN5_3_ in the inclusion lumen than M13-IN5_3_. This finding indicates that the phages successfully enter the host cells and that RGD enhances the uptake of phages by *C. t* and HeLa cells, while IN5 enhances localization in inclusion bodies.

### 3.5 Developmental stage-dependent effects of M13-IN53 and M13-RGD8-IN53 on C. t infection

**Fig. 5:**
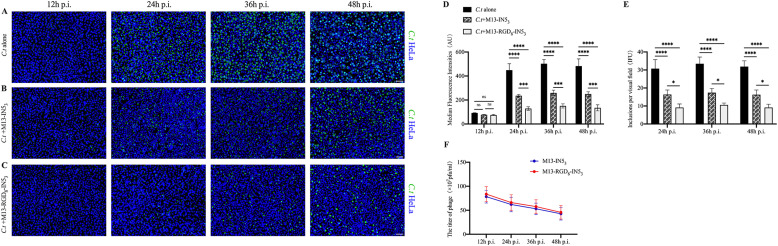
The effect of engineered phages on *C .t* infection at different time points p.i.: (A, B, C) Engineered phages are effective in reducing *C. t* infection in HeLa cells. HeLa cells were treated with M13-RGD_8_-IN5_3_ or M13-IN5_3_ phages (10^9^ pfu/mL) with *C. t* at a MOI of 1 (see Methods). (A): *C. t* infection alone control; (B): Pre-treatment with M13-IN5_3_; (C): Pre-treatment with M13-RGD_8_-IN5_3_; Scale bar = 50 μm; Blue: DAPI; Green: *C. t* inclusions. (D) The mean fluorescence intensity showed that pre-treatment with M13-IN5_3_ or M13-RGD_8_-IN5_3_ significantly reduced *C. t* infection in HeLa cells compared with that of *C. t* alone at 24, 36, and 48 h p.i. ****p < 0.0001. The mean fluorescence intensity was reduced in the M13-RGD_8_-IN5_3_ group compared to the M13-IN5_3_ group at 24, 36, and 48 h p.i. ***p < 0.0001. There was no difference at 12 h p.i. among the three groups (ns, *p* > 0.05). (E) Inclusion body counts showed that pre-treatment with M13-IN5_3_ or M13-RGD_8_-IN5_3_ significantly reduced *C. t* infection in HeLa cells compared with that of *C. t* alone at 24, 36, and 48 h p.i. More reduction of *C.t* inclusion numbers was found in the M13-RGD8-IN5_3_ group. **p < 0.01; ***p < 0.001; ****p < 0.0001. (F) The titer of phages at each time point p.i. Data represent the mean± standard deviation of three independent experiments.

To investigate the underlying mechanisms by which the recombinant M13-IN5_3_ and M13-RGD_8_-IN5_3_ phages affect *C. t* growth, we evaluated the inhibitory effects of the phages by immunofluorescence microscopy and qPCR during different stages of the *C. t* developmental cycle. We found that *C. t* fluorescence intensities were not significantly affected by phage infection at early time points (up to 12 h p.i.) compared with the *C. t* alone group (ns, *p* > 0.05, Fig. 5A–5D). *C. t* growth in the phage groups was inhibited at 24, 36, and 48 h p.i. (the mid and late stages of the *C. t* life cycle, *p* < 0.0001, Fig. 5D, 5E), indicated by a significant decrease in the number of inclusion bodies and fluorescence intensity. In addition, the inhibitory effects of M13-RGD_8_-IN5_3_ phages were significantly higher than those of M13-IN5_3_ (*p* < 0.001 for fluorescence intensities; *p* < 0.01 for inclusion numbers; Fig. 5D, 5E, Supplementary Table S3). To confirm the presence of phages in the HeLa cells at different *C. t* developmental stages, phage titers were quantified at 12, 24, 36, and 48 h p.i. We found that phages were present in the HeLa cells during the entire *C. t* development cycle (Fig. 5F).

**Fig. 6:**
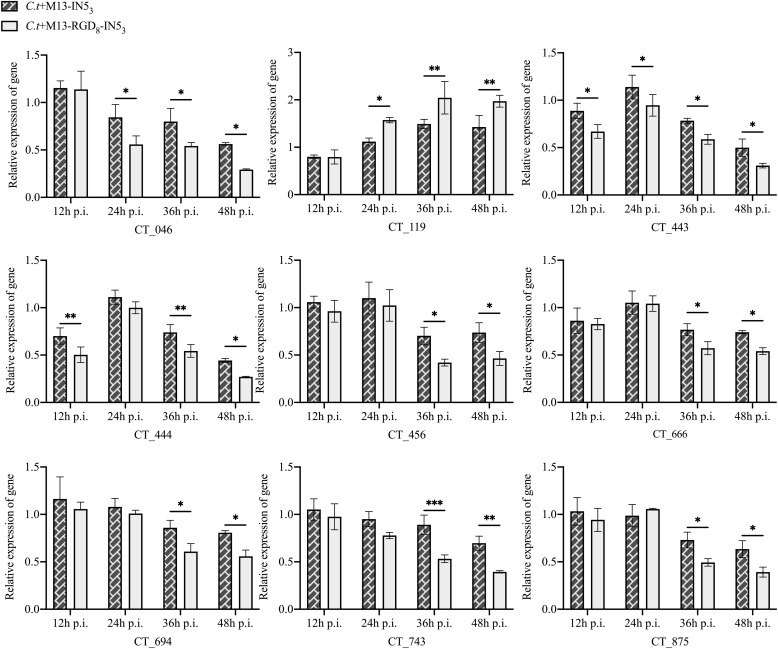
Total RNA was extracted from the infected cells at the indicated post-infection (p.i.) times and gene expression was tested by qPCR. The amplification efficiency of the target and housekeeping genes were nearly 100 %. If the relative expression quantity of a gene > 1, transcription was increased compared with the phage-free *C.t* group. If the relative expression quantity of a gene < 1, transcription was decreased for that gene. The expression of each gene in the M13-RGD_8_-IN5_3_ group was significantly different from that in the M13-IN5_3_ group. *p < 0.05; **p < 0.01. Data represent the mean ± standard deviation of three independent experiments.

We next performed qPCR on a selected subset of genes that are related to *C. t* virulence from 12 to 48 h p.i. in the M13-RGD_8_-IN5_3_- and M13-IN5_3_-treated and untreated *C. t* samples (Fig. 6). Only *CT_119* was upregulated in the M13-IN5_3_ and M13-RGD_8_-IN5_3_ groups at 36 h and 48 h p.i. compared with the phage-free group (relative gene expression >1, *p* < 0.05, Supplementary Table S4). *CT_046, CT_443, CT_444, CT_456, CT_666, CT_694, CT_743*, and *CT_875* were downregulated at 48 h p.i. compared with the phage-free group, but with differences in magnitude between the M13-IN5_3_ and M13-RGD_8_-IN5_3_ groups (relative gene expression <1, *p* < 0.05, Supplementary Table S4). Transcriptional changes in the *CT_443, CT_444*, and *CT_456* genes compared with the phage-free group at 12 h p.i. occurred without distinguishable fluorescence intensity changes (*p* > 0.05, Fig. 5D, and Supplementary Table S4), suggesting that the gene expression differences had not yet been translated into effects that were phenotypically observable. In contrast, differences in gene transcription at 24 h or later were associated with marked fluorescence intensity and inclusion number changes (*p* < 0.0001, Fig. 5D, 5E, and Supplementary Table S4). *CT_119* transcription in the M13-RGD_8_-IN5_3_ group was higher than that in the M13-IN5_3_ group (*p* < 0.05 at 24 h p.i.; *p* < 0.01 at 36 h p.i. and 48 h p.i., respectively, Fig. 6)*.* The transcript levels of all the downregulated genes in the M13-RGD_8_-IN5_3_ treatment group were lower than those in the M13-IN5_3_ group at 36 and 48 h p.i. (*p* < 0.05; *p* < 0.01; *p* < 0.01, Fig. 6).

## 4 Discussion

M13 is a member of the filamentous bacteriophage family. Filamentous bacteriophages have been used as vector-mediated therapeutic delivery methods in several tissues (Frenkel and Solomon, 2002). There are numerous reasons why the M13 phage is favorable for therapeutic delivery. First, it can be easily removed by the human body, without obvious side effects (Merril et al., 1996; Projan, 2004). Second, the M13 phage has high replication ability and can easily accommodate foreign DNA (Wen and Yuan, 2021). Peptides or proteins are often fused to the major capsid protein pⅧ and the minor capsid protein pIII. Compared with pⅧ, pⅢ allows for the insertion of protein sequences longer than 100 amino acids, making it more tolerant to foreign peptides than pⅧ. In addition, peptide insertion into pIII does not interfere with globular domain folding (Sidhu et al., 2000). In contrast, peptides fused into pⅧ often are only 6–8 amino acids long. Bhattarai et al. incorporated pmpD associated with *C. t* into the M13 phage and found that the phage was able to significantly reduce *C. t* infection in HeLa cells (Bhattarai et al., 2012). However, the construction of M13 phages incorporating antimicrobial proteins rather than *C. t*-associated proteins has not been well studied.

Although specific bacteriophages have been isolated from certain Chlamydia strains, there has been no evidence of *C. t* phages. We found that the Vp1 capsid protein of φCPG1 inhibits the growth of different *C. t* serovars (Ma et al., 2017). Furthermore, the IN5 loop of Vp1 ameliorated *C. t* infection, with an inhibition rate of 54.50 % (Zheng et al., 2017). In a previous study, we evaluated the effects of engineered M13-IN5_3_ phages on *C. t* infection. M13-IN5_3_ recombinant phages inhibited *C. t* growth. Indeed, the phages were able to enter the host cells and fill the cytosol, thus disrupting *C. t* growth, mainly in the developmental stage when RBs were converted into EBs (Lian et al., 2018). In the present study, to improve the inhibitory effect, we constructed an M13-RGD_8_-IN5_3_ recombinant phage (Fig. 1A). Our western blot data suggest that the phages successfully expressed high-density IN5 protein on pⅢ (Fig. 1B). Our specificity assay results indicate that the foreign peptide IN5 plays a direct role in interrupting intracellular *C. t* infection (Fig. 3A). We confirmed that the inhibitory effects of the recombinant phages were dose-dependent (Fig. 3B) and that there were no cytotoxic effects of the recombinant phages below or equal to 10^9^ pfu/mL in HeLa cells (Fig. 3C). In our study, we used PEG for the precipitation of phages, the PEG precipitation has drawbacks, the residual PEG content in the phage stocks may lead to cytotoxicity to HeLa cells (Branston et al., 2012). It is necessary to develop more effective purification systems for future research to reduce the PEG content. Furthermore, we speculated that high-titer recombinant phages expressing IN5 proteins may increase osmotic pressure which resulted in cell damage.

Based on these results, we chose a phage titer of 10^9^ pfu/mL for subsequent experiments. We determined that the recombinant phages could enter the HeLa cells and the *C. t* inclusion lumen by confocal fluorescence microscopy, and the permeability of recombinant phages with RGD was enhanced (Fig. 4). It is worth noting that the number of inclusions in the M13-RGD_8_-IN5_3_ and M13-IN5_3_ groups were reduced and the fluorescence intensity of *C. t* was weaker compared with the *C. t* infection-alone group, while the inhibitory rates in the group M13-RGD_8_-IN5_3_ were higher than those of M13- IN5_3_ (Fig. 5D, 5E, Supplementary Tables S2 and S3). Based on this result, we believe that the engineered M13-RGD_8_-IN5_3_ phage could be a better therapeutic than M13-IN5_3_ recombinant phages for *C. t* elimination due to the RGD motifs, which provide essential anchoring cues and thereby promote adhesion (Jeschke et al., 2002). Notably, we did not calculate the inclusion numbers at 12 h p.i. because the inclusion bodies were not mature during the early developmental cycle (Nicholson et al., 2003). There were no significant differences in the fluorescence intensity at 12 h p.i. between the M13-RGD_8_-IN5_3_ and M13-IN5_3_ groups (Fig. 5D)_._ The reduction in inclusion fluorescence intensity in M13-RGD_8_-IN5_3_ and M13-IN5_3_ phage groups at 24, 36, and 48 h p.i. suggests that they might reduce *C. t* replication within HeLa cells. Therefore, we speculate that the inhibitory effects of the recombinant phages on *C. t* might not occur in the early stages of the developmental cycle.

The invasive ability of EBs is critical for their infectivity. Our previous study observed abnormally enlarged RBs of *C. t* after Vp1 treatment. Further, Vp1 could arrest the chlamydial development cycle (Liu et al., 2012). In the present study, the EBs of *C. t* treated with recombinant phages were less infectious according to the progeny infectivity assay (Fig. 3B), which might be caused by the decrease in the EB amount or impaired invasive ability of EBs. Therefore, we investigated several genes related to EB infectivity during different stages of the *C. t* development cycle. qPCR analysis showed significant differences in the transcription of several chlamydial genes in the phage-treated groups compared to the control groups. At 48 h p.i., the relative expression of *CT_046, CT_443, CT_444, CT_456, CT_666, CT_694, CT_743,* and *CT_875* was significantly downregulated in phage-treated *C. t* (Fig. 6, Supplementary Table S4)*.* The only gene that was upregulated in the presence of phages was *CT_119* (*IncA*) (Fig. 6, Supplementary Table S4). Among them, *CT_119* (*IncA*) and *CT_443* (*OmcB*) are markers of mid- and late-stage chlamydial development (Petyaev et al., 2017).

IncA displays two coiled-coil domains that are critical in inhibiting *N*-ethylmaleimide-sensitive factor attachment receptor (SNARE)-mediated membrane fusion. Activation of IncA possibly prevents fusion with endocytic compartments, reduces host defense responses, and promotes homotypic inclusion membrane fusion (Delevoye et al., 2008; Ronzone and Paumet, 2013; Ronzone et al., 2014). In our study, we observed that *IncA* transcription in the phage groups was elevated at 36 and 48 h p.i. compared to the groups without phages (Supplementary Table S4). However, the number and fluorescence intensity of the inclusion bodies in the phage groups were lower at 36 and 48 h p.i. compared to those in the groups without phages (Fig. 6). We speculated that although *IncA* expression was elevated, the phages altered *IncA* translation, which led to abnormal coiled-coil domains that impaired function. However, this speculation needs to be confirmed by further research, as there is currently a lack of data about the effects of phages on the protein structure of IncA. The late-stage genes *CT_046* (*HctB*) and *CT_743* (*HctA*) encode lysine-rich proteins with a primary sequence similar to the eukaryotic histones *Hc1* and *Hc2*, which mediate DNA compression when RBs are converted into EBs (Mzobe et al., 2021). *HctA* and *HctB* are late-stage genes whose transcription occurs only late in the developmental cycle. We observed a decrease in the transcription levels of both genes at 36 and 48 h p.i., which may affect the formation of mature EBs with competent infectivity. Both omcA and omcB contain numerous cysteine residues, which may confer rigidity and osmotic integrity to chlamydial EBs (Hatch, 1996). Thus, decreased transcript levels of both *CT_444 (OmcA)* and *CT_443 (OmcB)* genes may affect the structural stability of EBs. *CT_456* (*Tarp*) is transcribed from the mid-to-late chlamydial developmental cycle (Belland et al., 2003). Tarp is a chlamydial type III secreted effector molecule and plays a role in the recruitment of actin to initiate *C. t* entry and internalization (Clifton et al., 2004). *C. t* lacking Tarp demonstrates significant attenuation of virulence in a murine genital tract infection model (Ghosh et al., 2020). *Cdsf (CT_666)* is concentrated in the outer membrane complex of EB, and *CT_666* encodes the chlamydial type three secretion system (T3SS) needle protein. T3SS is required for cell invasion, and numerous studies demonstrate that *CT_666* deletion or mutation results in significant attenuation of virulence (Betts et al., 2008). *CT_694 (TmeA)* plays an important role in T3SS-mediated invasion. *TmeA* is required during intravaginal infections because *TmeA* mutant strain infections in mice rapidly resolve (McKuen et al., 2017). Therefore, we concluded that the decreased transcriptional levels of *Tarp, Cdsf*, and *TmeA* in the phage groups may weaken the invasive ability of EBs during infection. As shown in Fig. 3B, the number of inclusions in the phage treatment groups was reduced. *CT_875* (*TepP*) plays a role in the invasion and/or establishment of nascent inclusions (Chen et al., 2014). In late-stage infection, the downregulation of *TepP* in the phage treatment groups may have negative effects on the invasion of *C. t* progeny. In addition, many immune-related genes, such as interferon-induced protein with tetratricopeptide repeats (IFITs), including IFIT1 and IFIT2, showed TepP-dependent activation. IFITs play a well-established role in host antiviral defense. Chen et al. (2014) found that the fold-change for IFIT1 and IFIT2 in the A2EN epithelial cells increased by 2-fold to more than 10-fold by 8 h p.i. with *C. t* L2 , which suggests that one of the functions of TepP is to modulate gene expression associated with innate immune responses in the early stages of infection. However, in our study, the transcriptional level of *TepP* was similar to that of the phage-free groups in the early stage of infection, which indicates that phage treatment might not affect host anti-bacterial defenses in the early infection stage. Whether decreased *TepP* transcription in the phage-treated groups in the middle-to-late *C. t* development cycle would affect the immune response needs further investigation.

According to the *C. t* gene expression results, the recombinant phages mainly suppress *C. t* development in the middle-to-late stages, similar to the effects of φCPG1 (Hsia et al., 2000; Śliwa-Dominiak et al., 2013). As a result, the development of mature EBs decreased their virulence. In addition, in our study, the transcriptional levels of all the genes mentioned above, except *CT_119* (*IncA*), in the M13-RGD_8_-IN5_3_ treatment group were lower than those in the M13-IN5_3_ treatment group, which was consistent with the results of better inhibitory effects of the recombinant M13-RGD_8_-IN5_3_ on the phenotypical level.

Our *in vitro* study cannot be used to draw conclusions regarding the use of our recombinant phages *in vivo*. The phage doses applied *in vivo* may be different because the internal environment *in vivo* is different from that *in vitro*. In addition, the immune system is able to eliminate foreign pathogens, including phages.

In summary, we successfully constructed recombinant M13-RGD_8_-IN5_3_ phages expressing the IN5 φCPG1 phage protein. We confirmed that M13-RGD_8_-IN5_3_ phages enter HeLa cells and inclusion bodies and inhibit *C. t* growth. The M13-RGD_8_-IN5_3_ phage was better than the M13-IN5_3_ phage in ameliorating *C. t* infection. The recombinant phages caused the downregulation of *C. t* genes related to infectivity, such as *CT_046* (*HctB*)*, CT_443* (*OmcB*), *CT_444* (*OmcA*), *CT_456* (*Tarp*), *CT_666* (*Cdsf*), *CT_694* (*TmeA*)*, CT_743* (*HctA*), and *CT_875* (*TepP*). The only upregulated gene was *CT_119* (*IncA*)*.* The recombinant phages had an impact on *C. t* mainly in the middle and late stages of the *C. t* developmental cycle. Our results suggest that novel recombinant phages are effective for treating *C. t* infection.

## Funding

This work was supported by the 10.13039/501100001809National Natural Science Foundation of China (grant 31870169 to QL), Science and Technology Project of 10.13039/501100010847Jiangxi Health Commission (grant No.: 202510462 to CY), Science and Technology research project of 10.13039/501100009102Jiangxi Education Department (grant No.: GJJ2401311 to CY), Young Scientists Fund of the 10.13039/501100004479Natural Science Foundation of Jiangxi Province (grant No.: 20242BAB20427 to CY). The funders had no role in study design, data collection and interpretation, or the decision to submit the work for publication

## Data availability statement

The original contributions presented in the study are included in the article/Supplementary Material; further inquiries can be directed to the corresponding authors.

## CRediT authorship contribution statement

**Cong You:** Writing – original draft, Methodology, Funding acquisition, Conceptualization. **Mei Wang:** Methodology, Data curation, Conceptualization. **Jiangyi Wang:** Writing – original draft, Methodology, Conceptualization. **Tingting Lian:** Data curation, Conceptualization. **Quanzhong Liu:** Funding acquisition, Conceptualization.

## Declaration of competing interest

The authors declare that they have no known competing financial interests or personal relationships that could have appeared to influence the work reported in this paper.

## Appendix Supplementary materials

Image, application 1

Image, application 2

Image, application 3

## References

[bib0001] Abedon S.T., Kuhl S.J., Blasdel B.G., Kutter E.M. (2011). Phage treatment of human infections. Bacteriophage.

[bib0002] Atterbury R.J. (2009). Bacteriophage biocontrol in animals and meat products. Microb. Biotechnol..

[bib0003] Belland R.J., Zhong G., Crane D.D., Hogan D., Sturdevant D., Sharma J., Beatty W.L., Caldwell H.D. (2003). Genomic transcriptional profiling of the developmental cycle of Chlamydia trachomatis. Proc. Natl Acad. Sci. U. S. A..

[bib0004] Betts H.J., Twiggs L.E., Sal M.S., Wyrick P.B., Fields K.A. (2008). Bioinformatic and biochemical evidence for the identification of the type III secretion system needle protein of Chlamydia trachomatis. J. Bacteriol..

[bib0005] Bhattarai S.R., Yoo S.Y., Lee S.W., Dean D. (2012). Engineered phage-based therapeutic materials inhibit chlamydia trachomatis intracellular infection. Biomaterials.

[bib0006] Branston S., Stanley E., Keshavarz-Moore E., Ward J. (2012). Precipitation of filamentous bacteriophages for their selective recovery in primary purification. Biotechnol. Prog..

[bib0007] Brockett M.R., Liechti G.W. (2021). Persistence alters the interaction between Chlamydia trachomatis and its host cell. Infect. Immun..

[bib0008] Chae S.Y., Shrestha K.R., Jeong S.N., Park G., Yoo S.Y. (2019). Bioinspired RGD-engineered bacteriophage nanofiber cues against oxidative stress. Biomacromolecules.

[bib0009] Chen Y.S., Bastidas R.J., Saka H.A., Carpenter V.K., Richards K.L., Plano G.V., Valdivia R.H. (2014). The Chlamydia trachomatis type III secretion chaperone Slc1 engages multiple early effectors, including TepP, a tyrosine-phosphorylated protein required for the recruitment of CrkI-II to nascent inclusions and innate immune signaling. PLOS Pathog.

[bib0010] Clifton D.R., Fields K.A., Grieshaber S.S., Dooley C.A., Fischer E.R., Mead D.J., Carabeo R.A., Hackstadt T. (2004). A chlamydial type III translocated protein is tyrosine-phosphorylated at the site of entry and associated with recruitment of actin. Proc. Natl Acad. Sci. U. S. A..

[bib0011] Delevoye C., Nilges M., Dehoux P., Paumet F., Perrinet S., Dautry-Varsat A., Subtil A. (2008). SNARE protein mimicry by an intracellular bacterium. PLOS Pathog.

[bib0012] Donnelly A., Yata T., Bentayebi K., Suwan K., Hajitou A. (2015). Bacteriophage mediates efficient gene transfer in combination with conventional transfection reagents. Viruses.

[bib0013] European Centre for Disease Prevention and Control (2025). STI cases on the rise across Europe. https://www.ecdc.europa.eu/en/news-events/sti-cases-rise-across-europe.

[bib0014] Frenkel D., Solomon B. (2002). Filamentous phage as vector-mediated antibody delivery to the brain. Proc. Natl Acad. Sci. U. S. A..

[bib0015] Ghosh S., Ruelke E.A., Ferrell J.C., Bodero M.D., Fields K.A., Jewett T.J. (2020). Fluorescence-reported allelic exchange mutagenesis-mediated gene deletion indicates a requirement for Chlamydia trachomatis Tarp during *in vivo* infectivity and reveals a specific role for the C terminus during cellular invasion. Infect. Immun..

[bib0016] Guo Y., Guo R., Zhou Q., Sun C., Zhang X., Liu Y., Liu Q. (2016). Chlamydiaphage φCPG1 capsid protein Vp1 inhibits Chlamydia trachomatis growth via the mitogen-activated protein kinase pathway. Viruses.

[bib0017] Hatch T.P. (1996). Disulfide cross-linked envelope proteins: the functional equivalent of peptidoglycan in chlamydiae?. J. Bacteriol..

[bib0018] Hsia R., Ohayon H., Gounon P., Dautry-Varsat A., Bavoil P.M. (2000). Phage infection of the obligate intracellular bacterium, Chlamydia psittaci strain guinea pig inclusion conjunctivitis. Microbes Infect.

[bib0019] Jeschke B., Meyer J., Jonczyk A., Kessler H., Adamietz P., Meenen N.M., Kantlehner M., Goepfert C., Nies B. (2002). RGD-peptides for tissue engineering of articular cartilage. Biomaterials.

[bib0020] Jian H., Chen Z.W., Yue X.L., Li J., Zhang J.H., Gong X.D. (2014). Epidemic trends and spatiotemporal distribution characteristics of genital Chlamydia trachomatis infection in China from 2018 to 2023 (in Chinese). Chin. J. Dermatol..

[bib0021] Jin H.E., Lee S.W. (2018). Engineering of M13 bacteriophage for development of tissue engineering materials. Methods Mol. Biol..

[bib0022] Kutter E., De Vos D., Gvasalia G., Alavidze Z., Gogokhia L., Kuhl S., Abedon S.T. (2010). Phage therapy in clinical practice: treatment of human infections. Curr. Pharm. Biotechnol..

[bib0023] Lang L.H. (2006). FDA approves use of bacteriophages to be added to meat and poultry products. Gastroenterology.

[bib0024] Lian T.T., Wei S.J., Liu Y.J., Ren J., Wang S., Guo Y.L., Guo R., Liu Q., Shao L. (2018). Construction and identification of the recombinant M13-IN5 phage and its effect on *Chlamydia trachomatis*. (in Chinese). Chin. J. Dermatol..

[bib0025] Liu Y.J., Hou S.P., Wei J.R., Li Y., Qi M.L., Wang H.P., Liu Q.Z. (2012). The effect of chlamydiaphage phiCPG1 capsid protein Vp1 on the Chlamydia trachomatis. (in Chinese). Chin. J. Microbiol. Immunol..

[bib0026] Ma J., Sun Y., Sun C., Zhou Q., Qi M., Kong J., Wang J., Liu Y., Liu Q. (2017). Identification of proteins differentially expressed by Chlamydia trachomatis treated with chlamydiaphage capsid protein VP1 during intracellular growth. Arch. Microbiol..

[bib0027] Manhart L.E., Gillespie C.W., Lowens M.S., Khosropour C.M., Colombara D.V., Golden M.R., Hakhu N.R., Thomas K.K., Hughes J.P., Jensen N.L., Totten P.A. (2013). Standard treatment regimens for nongonococcal urethritis have similar but declining cure rates: a randomized controlled trial. Clin. Infect. Dis..

[bib0028] McKuen M.J., Mueller K.E., Bae Y.S., Fields K.A. (2017). Fluorescence-reported allelic exchange mutagenesis reveals a role for Chlamydia trachomatis TmeA in invasion that is independent of host AHNAK. Infect. Immun..

[bib0029] Merril C.R., Biswas B., Carlton R., Jensen N.C., Creed G.J., Zullo S., Adhya S. (1996). Long-circulating bacteriophage as antibacterial agents. Proc. Natl Acad. Sci. U. S. A..

[bib0030] Mzobe G.F., Ngcapu S., Joubert B.C., Sturm W.A. (2021). Differential expression of groEL-1, incB, pyk-F, tal, hctA and omcB genes during Chlamydia trachomatis developmental cycle. PLoS One.

[bib0031] Nicholson T.L., Olinger L., Chong K., Schoolnik G., Stephens R.S. (2003). Global stage-specific gene regulation during the developmental cycle of Chlamydia trachomatis. J. Bacteriol..

[bib0032] Petyaev I.M., Zigangirova N.A., Morgunova E.Y., Kyle N.H., Fedina E.D., Bashmakov Y.K. (2017). Resveratrol inhibits propagation of Chlamydia trachomatis in McCoy cells. BioMed Res. Int..

[bib0033] Pranjol M.Z., Hajitou A. (2015). Bacteriophage-derived vectors for targeted cancer gene therapy. Viruses.

[bib0034] Projan S. (2004). Phage-inspired antibiotics?. Nat. Biotechnol..

[bib0035] Ren J., Lian T., Shao L., Liu Y., Liu Q. (2018). PmpI antibody reduces the inhibitory effect of Vp1 on Chlamydia trachomatis infectivity. Can. J. Microbiol..

[bib0036] Ronzone E., Paumet F. (2013). Two coiled-coil domains of Chlamydia trachomatis IncA affect membrane fusion events during infection. PLOS One.

[bib0037] Ronzone E., Wesolowski J., Bauler L.D., Bhardwaj A., Hackstadt T., Paumet F. (2014). An α-helical core encodes the dual functions of the chlamydial protein IncA. J. Biol. Chem..

[bib0038] Safari Z., Soudi S., Jafarzadeh N., Hosseini A.Z., Vojoudi E., Sadeghizadeh M. (2019). Promotion of angiogenesis by M13 phage and RGD peptide *in vitro* and *in vivo*. Sci. Rep..

[bib0039] Shao L., You C., Cao J., Jiang Y., Liu Y., Liu Q. (2020). High treatment failure rate is better explained by resistance gene detection than by minimum inhibitory concentration in patients with urogenital Chlamydia trachomatis infection. Int. J. Infect Dis..

[bib0040] Shin Y.C., Lee J.H., Jin L., Kim M.J., Oh J.W., Kim T.W., Han D.W. (2014). Cell-adhesive RGD peptide-displaying M13 bacteriophage/PLGA nanofiber matrices for growth of fibroblasts. Biomater. Res..

[bib0041] Sidhu S.S., Weiss G.A., Wells J.A. (2000). High copy display of large proteins on phage for functional selections. J. Mol. Biol..

[bib0042] Śliwa-Dominiak J., Suszyńska E., Pawlikowska M., Deptuła W. (2013). Chlamydia bacteriophages. Arch. Microbiol..

[bib0043] Stephens A.J., Aubuchon M., Schust D.J. (2011). Antichlamydial antibodies, human fertility, and pregnancy wastage. Infect. Dis. Obstet. Gynecol..

[bib0044] Tsevat D.G., Wiesenfeld H.C., Parks C., Peipert J.F. (2017). Sexually transmitted diseases and infertility. Am. J. Obstet. Gynecol..

[bib0045] Wen J., Yuan K. (2021). Phage display technology, phage display system, antibody library, prospects and challenges. AiM.

[bib0046] World Health Organization (2025). Chlamydia. https://www.who.int/news-room/fact-sheets/detail/chlamydia.

[bib0047] Yoo S.Y., Jin H.E., Choi D.S., Kobayashi M., Farouz Y., Wang S., Lee S.W. (2016). M13 bacteriophage and adeno-associated virus hybrid for novel tissue engineering material with gene delivery functions. Adv. Healthc. Mater..

[bib0048] Zheng L., Liu Y.J., Guo R., Zhou Q., Zhou W.J., Liu Q.Z. (2017). Cloning, expressing and identifying of IN5 part of chlamydiaphage phiCPG1 capsid protein Vp1 protein and its inhibitory effect on the *Chlamydia trachomatis*. (in Chinese). Chin. J. Clin. Infect. Dis..

