# Supplementary figures and images for: M13 phages engineered with chlamydia phage φCPG1 protein IN5 and arginine-glycine-aspartic acid inhibits Chlamydia trachomatis intracellular growth
Source: Virus Res. 2025 Oct 18;361:199645. doi: 10.1016/j.virusres.2025.199645 (PMC12593711; doi:10.1016/j.virusres.2025.199645)

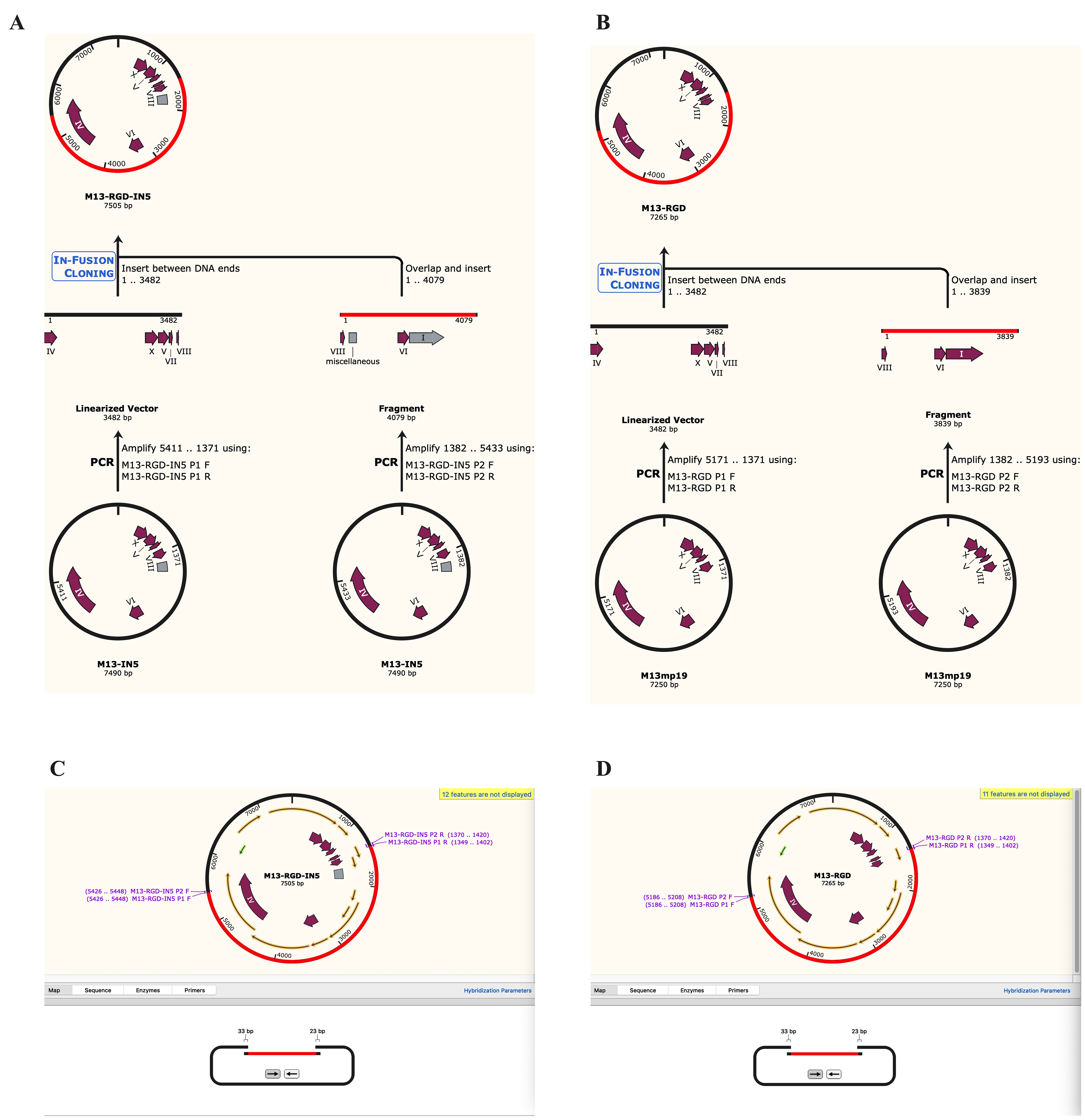

Supplement: Supplementary file 1 [file mmc1.zip › mmc1.tiff]

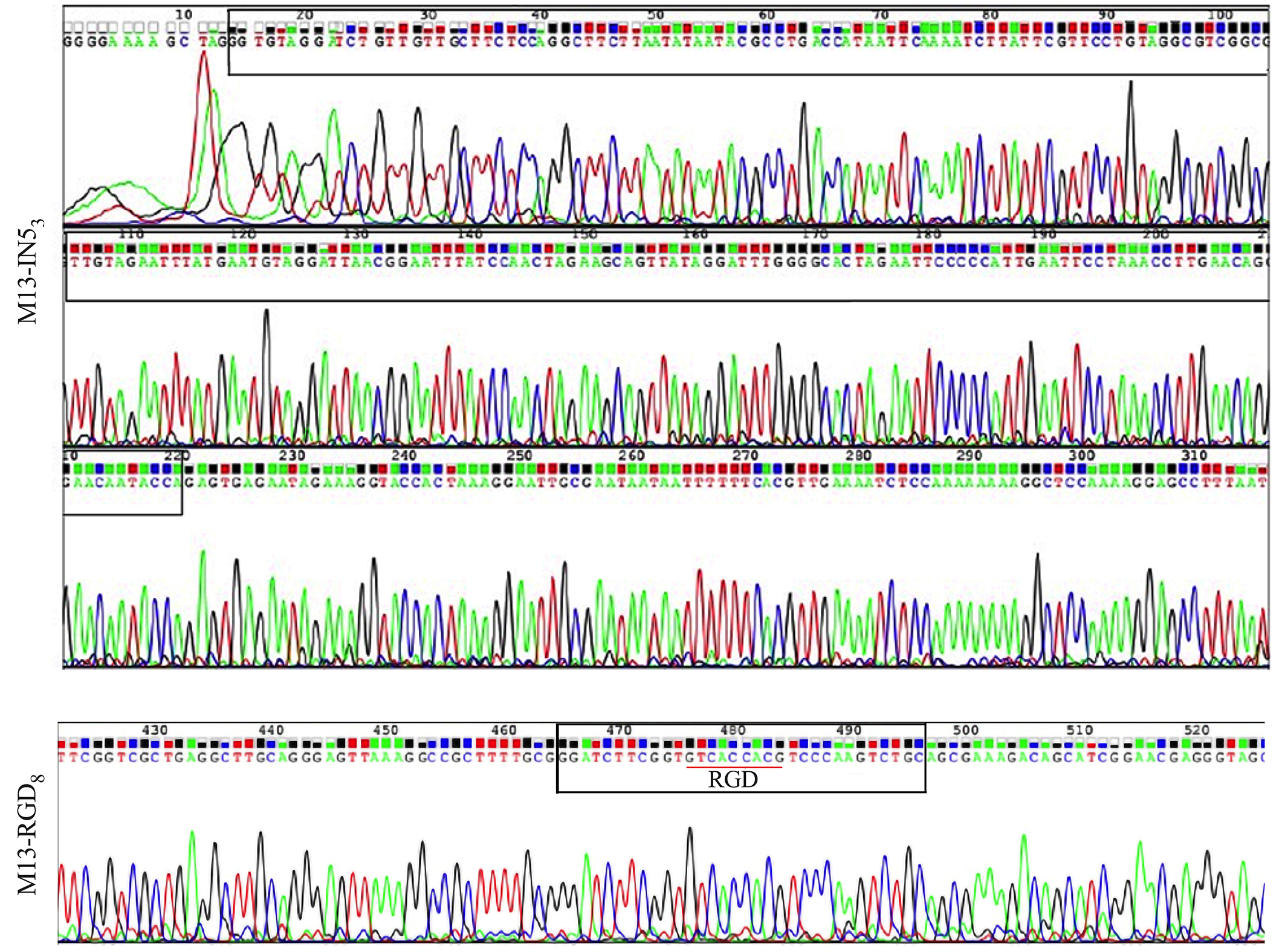

Supplement: Supplementary file 2 [file mmc2.zip › mmc2.tif]
