# Supplementary material for: M13 phages engineered with chlamydia phage φCPG1 protein IN5 and arginine-glycine-aspartic acid inhibits Chlamydia trachomatis intracellular growth
Source: Virus Res. 2025 Oct 18;361:199645. doi: 10.1016/j.virusres.2025.199645 (PMC12593711; doi:10.1016/j.virusres.2025.199645)
Supplement: Supplementary file 3 [file mmc3.docx]

Supplementary Material

# Supplementary Figures and Tables

**Supplementary Fig. 1.** Schematic diagram of genetic engineering of sequences for M13-RGD_8_-IN5_3_ and M13-RGD_8_ phage construction. The diagram was produced by SnapGene software 4.3.6 (Insightful Science Company, San Diego, CA, USA).


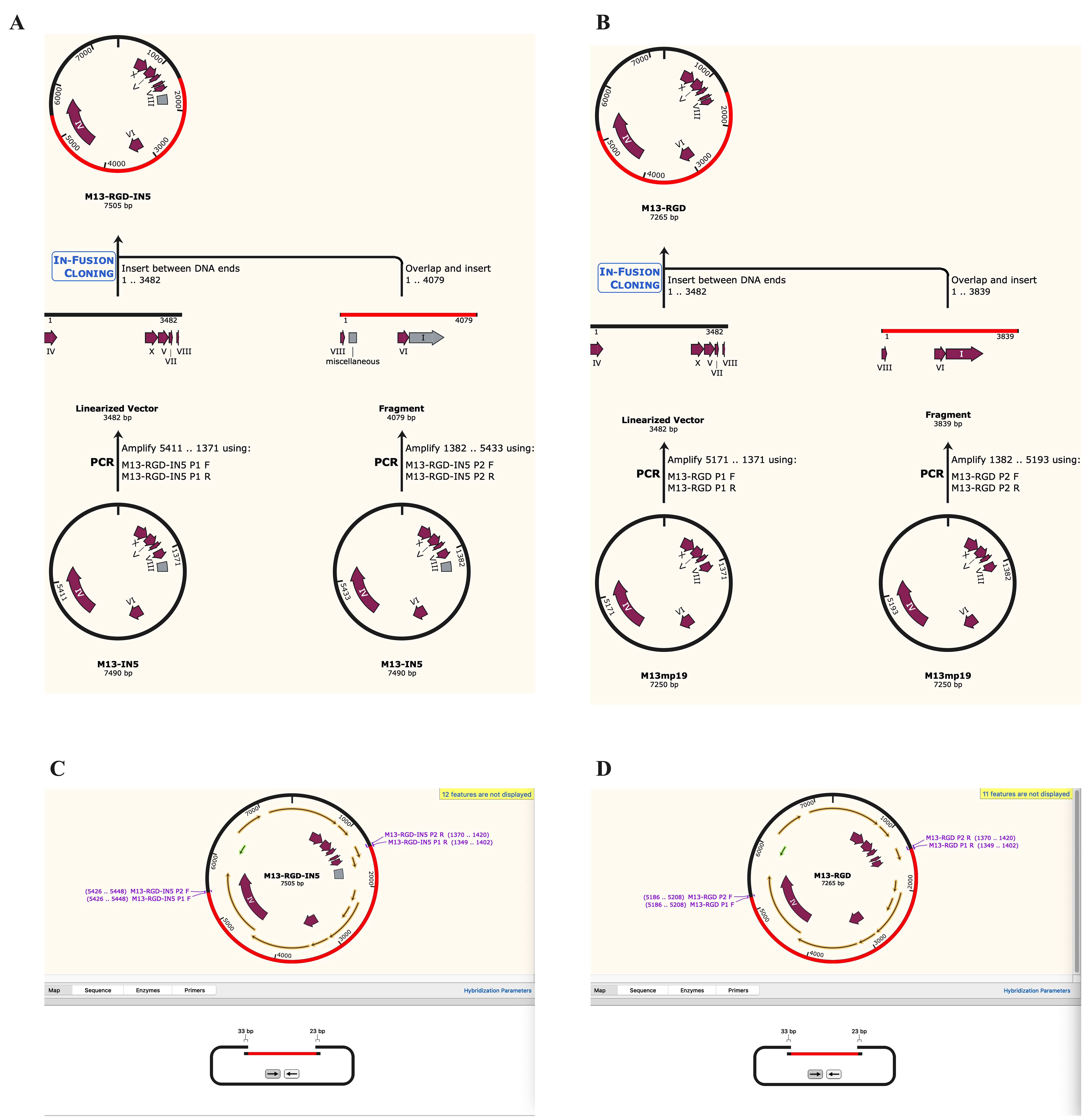


**Supplementary Fig. 2.** DNA sequence verification and locations of inserts in the M13 phage sequence. RGD peptide and IN5 protein were engineered on pⅧ and pⅢ, respectively.


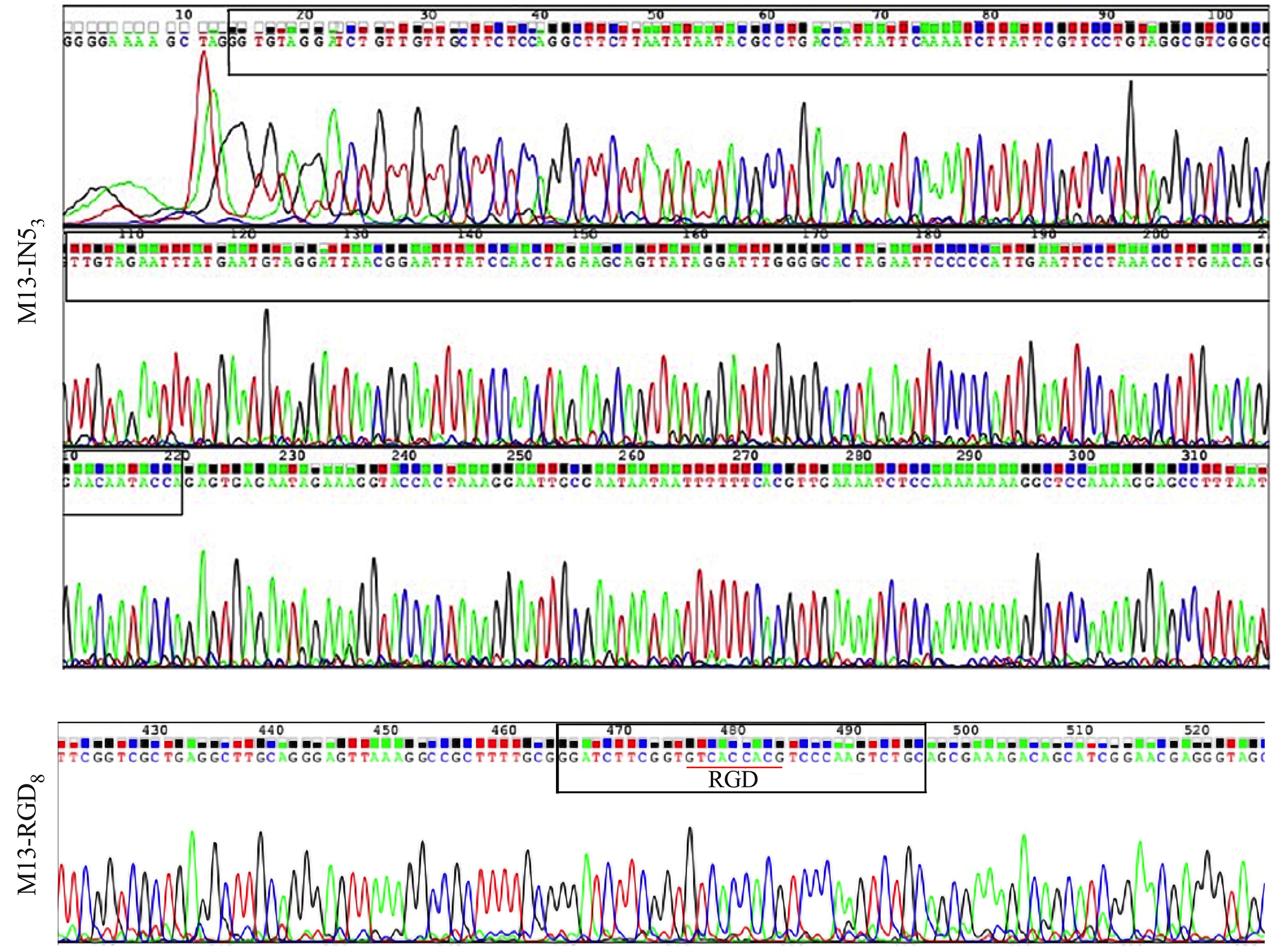


**Supplementary Table 1** Primer sequences of *Chlamydia trachomatis* genes used for qPCR.

| Gene name | Primer sequences (5' to 3') | Product length (bp) | Gene ID (NCBI) |
| --- | --- | --- | --- |
| *CT_r01 (16SrRNA)* | ACACCGCCCGTCACATCA | 97 | 884531 |
|  | ATCCTAGTCATCAGCCTCACCTTG |  |  |
| *CT_046 (HctB)* | GCTGCTTCTGGTGTAAAGGTTTGC | 87 | 884071 |
|  | TTGACGCCAACTGTGAGCTGTAC |  |  |
| *CT_119 (IncA)* | GAAAGCCAACAAGATGTCCCAAAAGAG | 136 | 884147 |
|  | GCACCTTCCTACTCAGCCAATCG |  |  |
| *CT_443 (OmcB)* | TGTCCAGAAGAATGAGCGTAACCATC | 118 | 884223 |
|  | CTTGTTTGCGTTGCCCAGTAGTTTAC |  |  |
| *CT_444 (OmcA)* | AACCACCGTCTACGTCTTTCTTCTTAG | 93 | 884216 |
|  | GTTGTCGTATCGTTGACTGTTGCTTC |  |  |
| *CT_456 (Tarp)* | CCTGGCGACAATGGTGGTTCTAC | 150 | 884231 |
|  | TCCTGCTGATTCCGTTGAAGAAGTG |  |  |
| *CT_666 (Cdsf)* | AACATCCACGCAAGGAACTGTAGAC | 110 | 884451 |
|  | TCACCGCAGTCAAGATATTGGATACAG |  |  |
| *CT_694 (TmeA)* | CGCAGAATAAACAGACTTTGGCTACAC | 127 | 884482 |
|  | TCGCCTGGAGTAAACAGCATCAATC |  |  |
| *CT_743 (HctA)* | ACAGGCTTCTTAGCAGCAGCTTTAG | 149 | 884538 |
|  | GCAGCACAAAGAGTTCGTACAGAATC |  |  |
| *CT_875 (TepP)* | GATTCGCCGTTGCTTCTCTTGTTG | 134 | 884145 |
|  | CACCTCGCTCTATTCCCATATCATCTG |  |  |

**Supplementary Table 2** Inclusion body inhibition by M13-IN5_3_ and M13-RGD8-IN5_3_ at different titers.

| Titers of phages (pfu/mL) | M13-IN5_3_ (%) | M13-RGD_8_-IN5_3_ (%) | |
| --- | --- | --- | --- |
| 10^6^ | 11.50 ± 3.63 | | 11.04 ± 2.98 |
| 10^7^ | 25.47 ± 5.93 | | 33.48 ± 2.36 |
| 10^8^ | 36.44 ± 7.09 | | 52.00 ± 1.85 |
| 10^9^ | 48.09 ± 6.85 | | 69.79 ± 3.71 |

**Supplementary Table 3** Inhibitory rates of inclusion bodies and fluorescence intensity by M13-IN5_3_ and M13-RGD_8_-IN5_3_ at a titer of 10^9^ pfu/mL at different times post-infection.

| Hours post  infection | Inclusion bodies | |  | Fluorescence intensity | | |
| --- | --- | --- | --- | --- | --- | --- |
|  | M13-IN5_3_ (%) | M13-RGD_8_-IN5_3_ (%) |  | M13-IN5_3_ (%) | M13-RGD_8_-IN5_3_ (%) | |
| 24 | 46.66 ± 3.96 | 70.44 ± 1.58 |  | 47.21 ± 4.58 | | 71.26 ± 1.24 |
| 36 | 48.15 ± 1.39 | 68.35 ± 0.34 |  | 48.51 ± 1.11 | | 70.11 ± 1.74 |
| 48 | 49.14 ± 3.12 | 71.55 ± 2.98 |  | 48.04 ± 2.73 | | 72.23 ± 2.02 |

**Supplementary Table 4** Analysis of gene transcription fold changes at different hours post infection in different phage groups (2*^−ΔΔct^*)

| Gene name | M13-IN5 | | | |  | M13-RGD-IN5 | | | |
| --- | --- | --- | --- | --- | --- | --- | --- | --- | --- |
|  | 12 h p.i. | 24 h p.i. | 36 h p.i. | 48 h p.i. |  | 12 h p.i. | 24 h p.i. | 36 h p.i. | 48 h p.i. |
| *CT_046* | 1.15 ± 0.08 | 0.84 ± 0.13 | 0.80 ± 0.14* | 0.56 ± 0.02* |  | 1.14 ± 0.19 | 0.56 ± 0.09* | 0.54 ± 0.04* | 0.29 ± 0.01* |
| *CT_119* | 0.80 ± 0.04 | 1.12 ± 0.07 | 1.49 ± 0.10* | 1.43 ± 0.25* |  | 0.79 ± 0.15 | 1.57 ± 0.06* | 2.04 ± 0.34* | 1.97 ± 0.12* |
| *CT_443* | 0.89 ± 0.08 | 1.14 ± 0.13 | 0.78 ± 0.03* | 0.50 ± 0.09* |  | 0.67 ± 0.07* | 0.95 ± 0.11 | 0.59 ± 0.05* | 0.31 ± 0.02* |
| *CT_444* | 0.70 ± 0.09* | 1.11 ± 0.07 | 0.74 ± 0.08* | 0.44 ± 0.02* |  | 0.50 ± 0.08* | 1.00 ± 0.06 | 0.54 ± 0.07* | 0.27 ± 0.01* |
| *CT_456* | 1.06 ± 0.06 | 1.10 ± 0.17 | 0.70 ± 0.09* | 0.74 ± 0.10* |  | 0.96 ± 0.12 | 1.02 ± 0.17 | 0.42 ± 0.04* | 0.46 ± 0.07* |
| *CT_666* | 0.86 ± 0.13 | 1.05 ± 0.12 | 0.77 ± 0.06* | 0.74 ± 0.02* |  | 0.83 ± 0.06* | 1.04 ± 0.08 | 0.57 ± 0.07* | 0.54 ± 0.04* |
| *CT_694* | 1.16 ± 0.23 | 1.08 ± 0.09 | 0.86 ± 0.08 | 0.81 ± 0.02* |  | 1.06 ± 0.07 | 1.01 ± 0.04 | 0.61 ± 0.08* | 0.56 ± 0.07* |
| *CT_743* | 1.05 ± 0.11 | 0.95 ± 0.08 | 0.89 ± 0.10 | 0.70 ± 0.07* |  | 0.98 ± 0.14 | 0.78 ± 0.03* | 0.53 ± 0.04* | 0.39 ± 0.01* |
| *CT_875* | 1.03 ± 0.15 | 0.99 ± 0.12 | 0.73 ± 0.08* | 0.63 ± 0.09* |  | 0.94 ± 0.12 | 1.06 ± 0.01 | 0.49 ± 0.04* | 0.39 ± 0.05* |

* The expression of this gene in the phage group was significantly different from that in the *C.t* group (*p* < 0.05). If the fold change >1, transcription is increased for that purpose gene, conversely, if the fold change <1, transcription is decreased for that purpose gene. p.i.: post infection.
